# Report of a Case—Death in a Few Hours—What Was It

**Published:** 1886-03-20

**Authors:** J. A. Andrey

**Affiliations:** Pineville, North Carolina


### Southern Medical Record.

A MONTHLY JOURNAL OF PRACTICAL MEDICINE.

☞All Communications must be addressed to R. C. WORD, M. D., Managing Editor.

"*Quicquid Præcipies Esto Brevis.*"

VOL. XVI. ATLANTA, GA., MARCH 20, 1886. No. 3.

### ORIGINAL AND SELECTED ARTICLES.

### REPORT OF A CASE.—DEATH IN A FEW HOURS.—WHAT WAS IT?

BY J. A. ANDREY, M. D., PINEVILLE, N. C.

*Editors Southern Medical Record, Atlanta, Ga.*

GENTLEMEN: I herewith forward to you the following report of a case, and request that you, or any of your valuable contributors, impart to me any information you may feel inclined to do. The case is as follows:

The patient, a child two years old, extremely fat and healthy; a male. The history of the case, as given by its parents, is as follows: The patient was as well as usual on the morning of February 1. The parents report that the little fellow was somewhat restless on the night previous, and talked a little incoherently, but this condition of things lasted only a short while, and the patient slept soundly the balance of the night. The parents attributed its restlessness to a dose of sulp. quinia, which had been given at bedtime. The little patient awoke bright and lively on the morning of February 1, ate breakfast, and went off to play, as usual. The father and myself were absent The patient was suffering from an attack of coryza, but the attack was so mild as scarcely to attract attention; continued to play, and seemed perfectly well up to 1 o'clock p. m., when he showed some symptoms of ordinary chill; was a little cold; trembled some. All at once the child began to scream, and would throw itself backward and then forward; its mother thought that, as he had eaten a piece of raw turnip that morning, it was an ordinary attack of colic, and consequently resorted to such domestic remedies, as bathing in hot water, application of hot cloths to abdomen, gave a little whisky, etc. The child quit screaming and, as his mother supposed, went to sleep; but she soon discovered that his color and breathing were not right. She became alarmed and summoned medical aid immediately. The doctor arrived about half-past 2 p. m., and, as I learn from him, found the child's condition about as follows: Color of face deep livid, surface of body cool, pupil contracted, pulse quick and feeble, respiration of a peculiar spasmodic character, almost noiseless, not croupous or stridulous, but something like we see when the mouth and throat are full of mucous; totally unconscious; entirely insensible to pain; in fact, the child presented all the symptoms of profound coma.

I saw the case about 4 p. m., or about three hours after the onset of the attack. When turned over on his face, a white frothy mucous would issue from the child's mouth and nose, its pulse would fluctuate—be strong for a few moments, then almost cease; the respiration was exactly of that rattling, tearing character that is produced by air passing through mucous in mouth, throat, and bronchial tubes. Now, Messrs. Editors, I am an obscure village practitioner, and desire information and instruction. What was the matter with this little patient? Was it a case of suffocating catarrh; or a violent attack of congestion of brain, lungs, etc.? Was the attack the result of embolism, or was it spasm of the glottis; or what? Could the child have been choked? There was no struggling in order to get its breath, like we would expect to see if there had been a foreign body in its air passages. To make the case still plainer, there was complete insensibility of the entire cutaneous surface, so that sinapisms, blisters, etc had no appreciable effect whatever, not even reddening the surface.

Messrs. Editors, I will give you my theory, and I want you to tell me whether I am right or wrong. I think the patient had a chill at 1 p. m., when a violent attack of congestion of the lungs ushered in. The bronchial tubes were filled with mucous, producing, by mechanical pressure and a want of properly decarbonized blood, paralysis of the muscles of respiration, and, subsequently, of the entire system. Going on the theory that there was paralysis, the child could not swallow, we gave brandy hypodermically and by enema, quinine same way, hot baths, mustard and blisters; atropia hypodermically, and under the influence of atropia the surface reddened and pupil dilated, but the pulse remained feeble.

We resorted to everything usual in such cases to produce reaction, but we gained nothing. The patient grew worse rapidly, and died at 10 p. m.—nine hours from date of attack.

I once treated a similar case to above, and with exactly same result—child taking sick and dying in about same length of time. I was called to see a fat, chubby little baby once, suffering from a violent attack of croup, and found it in almost a similar condition ; but I arrived in a few moments after inception of the attack, and prompt and thorough emesis relieved the little sufferer. We tried to produce emesis in the above case, but failed signally. We tried running a trocar down its throat, and then a feather, and if I had had any apomorphia would have injected it hypodermically. I think, if we could have vomited the patient thoroughly, and thus have emptied the bronchial tubes, the patient would have been relieved, but this we could not do.

I have tried to make the case plain. What was the propor diognosis ?

[OUR first impression, on reading the above, was that the case was one of Cerebro-spinal Meningitis. Loomis, in the late edition of his work on Practice, says of this disease, that "The invasion is sometimes abrupt ; the patient, apparently in perfect health, is suddenly seized with a chill, loss of consciousness, becomes comatose, and dies in a few hours." During the last summer, when the disease was prevailing in this vicinity, a boy was suddenly seized with headache, rapidly became comatose, with evidences of violent congestion in the base of the brain, overwhelming the respiratory center in the medulla oblongata, causing pulmonary congestion, impeding respiration, and decarbonization of the blood, causing a livid appearance of the lips, and large dark ecchymosed spots on the face and other parts of the body, and the patient died within a few hours after the onset of the attack. In such cases the patient dies in the congestive stage of the attack, and before inflammatory action has supervened. While this is true of cerebro-spinal meningitis, cases like the above sometimes result from malarial influence, or from the increased susceptibility caused by teething, or both. Under such circumstances, an irritation, as from a piece of raw turnip or other crude article in the alimentary canal, may develop a chain of reflex phenomena like to those above described, attended with a like speedy and fatal result.—EDITORS ]

---

Dr. A. Walker says, in the British Medical Journal: "In a number of cases, strychnine administered along with iron for a month before labor, has exerted a remarkable influence in preventing *post partum* hemorrhage, where severe flooding has occurred in previous labor."—*Obstetric Gazette.*

